# PAIN MEDICATION AND FALL RISKS AMONG COMMUNITY-DWELLING OLDER ADULTS WITH ARTHRITIS

**DOI:** 10.1093/geroni/igad104.2564

**Published:** 2023-12-21

**Authors:** Aya Yoshikawa, Richard Fortinsky

**Affiliations:** Texas Woman’s University, Denton, Texas, United States; University of Connecticut, Farmington, Connecticut, United States

## Abstract

Pain medication is commonly used among older adults with arthritis. Research indicates an elevated fall risk among older adults with arthritis, yet this association among those who take medication for pain is not fully studied. This population-based study investigated the association of falls with pain medication use by arthritis status. A sample of community-dwelling Medicare beneficiaries in the 2015 National Health and Aging Trends Study, representing older adults who reported arthritis (n = 4,229) and no arthritis (n = 3,258), were analyzed using survey-weighted logistic regression analysis. Sensitivity analysis was performed to investigate differences in associations by the frequency of taking pain medication. All analyses were controlled for sociodemographic characteristics, living arrangements, balance problems, and chronic conditions. The majority of participants was 70 years or older (70.7%), female (55.3%), White (80.1%), had a high school or higher education (58.4%) and had two or more chronic conditions (53.6%). Significantly higher proportions of those with arthritis reported pain (67.9% vs. 32.1%) and pain medication use (63.1% vs. 36.9%) than those with no arthritis. Pain medication use (OR = 1.11, 95% CI: 1.03, 1.20) and balance problems (OR = 4.39, 95% CI 3.45, 5.59) were associated with falls among those with arthritis. Taking medication for pain every day a week (OR = 1.48, 95% CI: 1.05, 2.09) was associated with falls among those with arthritis. Findings suggest that the heavy use of pain medication may increase fall risk. Nonpharmacological pain management education is encouraged for supporting active living among older adults with arthritis.

